# Incarcerated peri-inguinal hernia containing the appendix: a case report

**DOI:** 10.1093/jscr/rjae847

**Published:** 2025-02-17

**Authors:** Julian M Baumkirchner, Mustafa Aydin, Sema Simoes de Almeida, Michael Zünd

**Affiliations:** Department of Surgery, Zuger Kantonsspital, Landhausstrasse 11, CH-6340 Baar, Switzerland; Department of Surgery, Zuger Kantonsspital, Landhausstrasse 11, CH-6340 Baar, Switzerland; Department of Surgery, Zuger Kantonsspital, Landhausstrasse 11, CH-6340 Baar, Switzerland; Department of Surgery, Kantonsspital Baden, Im Ergel 1, CH-5404 Baden, Switzerland; Department of Surgery, Zuger Kantonsspital, Landhausstrasse 11, CH-6340 Baar, Switzerland

**Keywords:** peri-inguinal hernia, incarcerated appendix, ventral hernia, abdominal wall abscess, case report

## Abstract

Primary ventral hernia (PVH) is a main differential diagnosis of abdominal wall masses and typically occurs at areas of structural weakness, such as the linea alba. This report draws attention to atypical PVHs of the peri-inguinal region, an underdiagnosed subgroup of defects located adjacent to the inguinal canal and below the semilunar line. The absence of a standardized definition or classification complicates the diagnosis of this infrequent pathology. We present a case of a patient with a tender abdominal wall mass, initially interpreted as superinfected hematoma. Despite drainage, the patient’s physical condition continued to deteriorate. Repeat imaging eventually revealed acute appendicitis inside a peri-inguinal hernia. Laparoscopic appendectomy was performed, and the abscess cavity was incised, debrided and left open, which led to successful secondary closure of the wound. This case highlights the need for greater awareness of atypical hernias and their potential complications to enable timely detection and adequate treatment.

## Introduction

With an estimated prevalence of 5% in the general population, abdominal wall defects can frequently be encountered in daily surgical practice. Ventral hernias are defects of the anterolateral abdominal wall, and must be differentiated from inguinal, femoral or diaphragmatic hernias. Based on etiology, primary hernias can be distinguished from incisional hernias, which develop after preceding surgical intervention [1]. Primary ventral hernias (PVHs) have been classified by the European Hernia Society according to their localization into midline and lateral hernias. Lateral hernias are further divided into Spigelian and lumbar hernias, indicating the corresponding area of ultrastructural weakness of the abdominal wall [2].

Cases of lateral PVH in regions other than those previously mentioned have only been reported sporadically, and little is known about their clinical significance [3–6]. Therefore, we present a case of atypical PVH of the peri-inguinal region complicated by incarceration of the appendix and discuss challenges in diagnosing and managing this exceptional pathology. This report follows the SCARE 2020 guidelines.

## Case report

**Figure 1:**
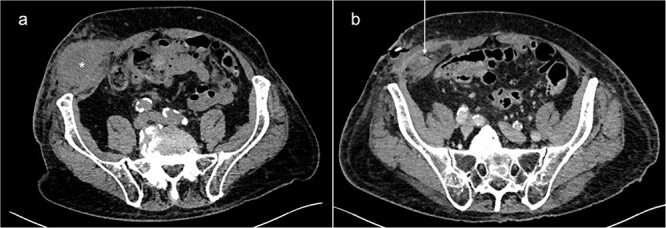
Abdominal CT-scans on initial presentation (a) and day 5 (b). A large fluid collection (*) can be seen in the right lower abdominal wall. After drainage, the appendix (white arrow) could be identified inside the abscess cavity, indicating the existence of a PVH.

An 87-year-old male on oral anticoagulant therapy was admitted to the emergency department with abdominal pain for 1 week. On physical examination, a painful mass could be palpated in the right lower quadrant of the abdomen, accompanied by local signs of inflammation. Blood tests revealed elevated levels of C-reactive protein (CRP) and white blood cells (WBC). The patient had no history of previous surgery in the lower abdomen. A computed tomography (CT) scan showed a large fluid collection in the abdominal wall, primarily interpreted as a superinfected hematoma (Fig. 1). Incision and drainage of the collection was performed, which released large quantities of pus. Consequently, the patient was put on broad-spectrum antibiotics.

**Figure 2:**
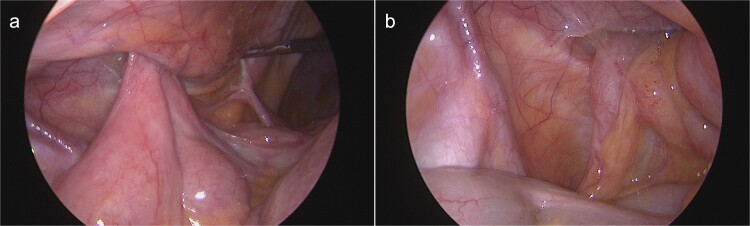
Intraoperative view on the hernia orifice containing terminal ileum (a) and the appendix (b).

**Figure 3:**
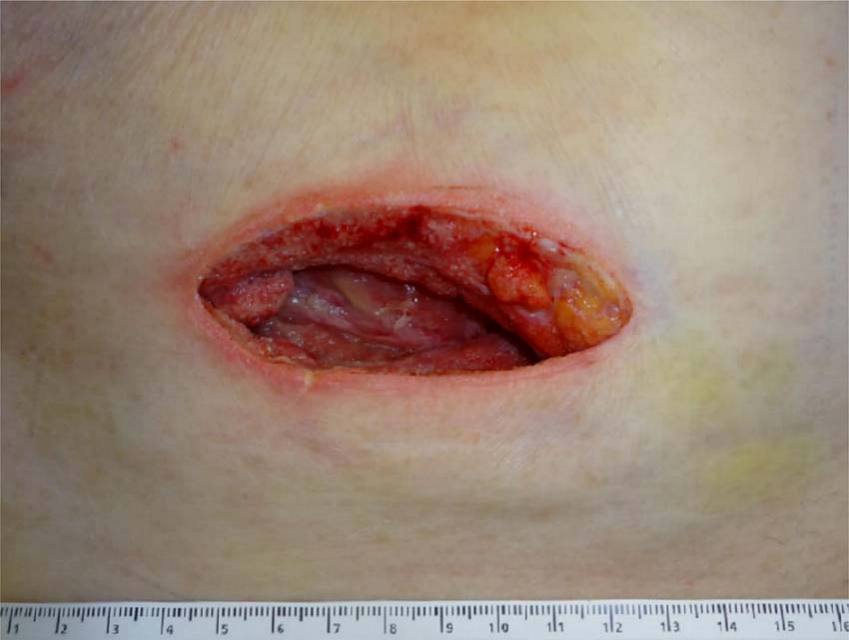
The abscess wound 2 days after application of a negative-pressure wound therapy device.

During the following days, purulent discharge from the wound continued and the patient developed signs of sepsis. Abdominal CT was repeated, which now revealed acute appendicitis inside an atypical PVH in the right peri-inguinal region (Fig. 1). Thus, the patient was taken back to the operating room for laparoscopy and abdominal wall exploration. Intraoperatively, a segment of terminal ileum and the appendix were found trapped inside the hernia orifice (Fig. 2). After reduction of the herniated contents, laparoscopic appendectomy was performed uneventfully. The remaining abscess cavity was generously incised, debrided and left open to facilitate secondary wound closure (Fig. 3). This was eventually achieved using a vacuum-assisted closure (VAC) device and secondary suture. After management of concomitant medical conditions, the patient was discharged from the hospital on the 11th postoperative day. No physical follow-up was conducted as the patient declined further surgery for hernia repair due to poor general condition.

## Discussion

Masses of the anterolateral abdominal wall can be caused by a variety of common pathologies, including lipoma, hematoma or solid tumors [7]. In the lower abdomen, Spigelian hernia (SpH) should be considered as a differential diagnosis. Named after the Belgian anatomist Adriaan van den Spieghel (1578–1625), it occurs through a defect in the Spigelian aponeurosis, which extends from the edge of the rectus abdominis muscle medially to the semilunar line laterally. Typically, SpH develops within a 6 cm wide band above the interspinal line, where the aponeurosis is largest and the abdominal pressure greatest. The hernia may cause intermittent pain and swelling sensation in the abdominal wall, often accompanied by an inconspicuous clinical examination [8].

SpH accounts for only 1%–2% of ventral hernias but carries a significant incarceration risk, reported as high as 24%. This is caused by the small hernia orifice, normally not exceeding 2 cm in diameter. Small intestine, greater omentum or colon are typically found within the hernia sac [8]. Incarcerated appendices have been reported occasionally. In several cases [9–12] abscess formation around the inflamed appendix was described, similar to what was observed in this case.

**Figure 4:**
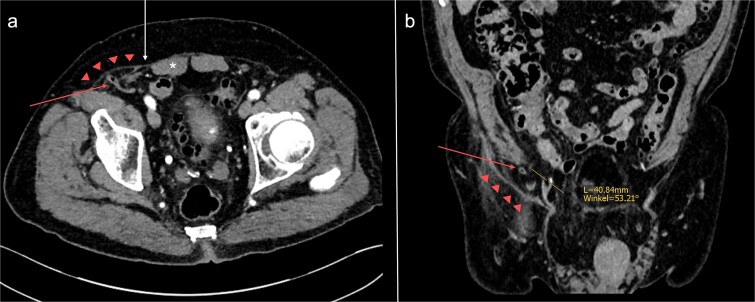
Abdominal CT-scan conducted 2 years earlier on the same patient, axial (a) and coronal (b) slices. The appendix (red arrow) is passing directly through the transversus abdominis and internal oblique muscles into the hernia sac (red triangles). The orifice is located ⁓4 cm superolateral to the right internal inguinal orifice (yellow line). *: rectus abdominis muscle; white arrow: Spigelian aponeurosis.

For this reason, incarcerated SpH was initially considered the patient’s primary diagnosis. However, previous CT-studies and intraoperative findings indicated that the hernia was not situated within the Spigelian aponeurosis. It had formed directly through the transversus abdominis and internal oblique muscles, ⁓4 cm superolateral to the internal inguinal ring, away from the myopectineal orifice (Fig. 4). Therefore, the defect does not comply with the definitions of Spigelian, lumbar, or inguinal hernia [2, 13]. It can, however, be classified as a so-called peri-inguinal hernia.

Para- and peri-inguinal hernias are located adjacent to the inguinal canal, below the semilunar line and beneath the aponeurosis of the external oblique muscle. Para-inguinal hernias, which penetrate the inguinal canal, can be distinguished from peri-inguinal hernias, which do not [14]. Historically, para-inguinal hernias were first mentioned by La Chausse in 1746. In his description of ventral hernias, he commented on parainguinal, medial-inguinal and supravesical hernias, noting that “No certain locus can be assigned to them.” [15]. In 1922, Holloway published an article on a hernia located 8 cm superolateral to the right internal inguinal orifice [3], which may be considered one of the first well-documented cases of this rare pathology. Over the subsequent decades, several reports of similar, atypical hernias of the peri-inguinal region followed [4]. Despite Gallese’s efforts to clearly define this hernia type in 1991 [14], his classification has not been widely adopted and, recently, other terms have been proposed to better describe these defects [4, 5]. At this point, we would like to emphasize the importance of including peri-inguinal hernias in established hernia classification systems, to avoid misclassification and facilitate research in this unexplored field of abdominal wall surgery.

Regarding treatment, open and laparoscopic mesh repair are currently used for the closely related Spigelian hernia [16]. In recently published papers on peri-inguinal hernia [4–6], laparoscopic mesh repair has been the predominant method of treatment. Mourad and Kharbutli described a patient with a large peri-inguinal defect containing preperitoneal fat, which could be covered with mesh using a total extraperitoneal approach [6]. Veréb-Amolini *et al*. reported a case of peri-inguinal hernia containing small intestine and adipose tissue in a patient with polycystic kidney disease and intracranial aneurysm, which was treated similarly [4]. Weber Sánchez and Palmisano described two cases of peri-inguinal hernia, both of which were repaired in transabdominal preperitoneal-type procedures [5].

Unlike previous reports, this case of peri-inguinal hernia was complicated by an incarcerated appendix. Samsami *et al*. reported a similar case of atypical ventral hernia containing the appendix in 2023 [17]. However, the associated defect was located more cranially and can therefore not be labeled peri-inguinal hernia. We chose to perform laparoscopic appendectomy but did not undertake hernia repair due to severe, purulent infection and the patient’s poor general condition. A comparable approach was selected by Peeters *et al*. in a patient with incarcerated Spigelian hernia [9]. In other cases of ventral hernia with incarcerated appendix [10–12, 17], appendectomy with simultaneous direct hernia repair has been performed successfully. It is noteworthy that all aforementioned cases abstained from the use of synthetic mesh for hernia repair, as this has been associated with high postoperative infection rates in contaminated surgical fields [18].

## Conclusion

PVHs should be considered as a differential diagnosis of lateral abdominal wall masses. Critical reevaluation can help to identify their presence. Peri-inguinal hernia is a poorly defined subcategory of PVHs, which lacks recognition in established hernia classifications. As demonstrated, peri-inguinal hernias can lead to incarceration of viscera, requiring timely surgical intervention. Awareness of the existence of atypical PVHs may reduce diagnostic error and enable early effective treatment.
